# Prevalence of Covid-19 Associated Symptoms, Their Onset and Duration, and Variations Among Different Groups of Patients in Bangladesh

**DOI:** 10.3389/fpubh.2021.738352

**Published:** 2021-09-29

**Authors:** Md. Tanzilul Amin, Mahmud Hasan, N. M. Mahmudul Alam Bhuiya

**Affiliations:** ^1^Department of Pharmacy, Jagannath University, Dhaka, Bangladesh; ^2^Department of Radiology and Radiological Science, Johns Hopkins University, Baltimore, MD, United States

**Keywords:** COVID-19, SARS-CoV-2, COVID-19 symptoms, corona, flu, symptoms, fever, cold

## Abstract

**Objective:** This study aimed to assess the prevalence, onset, and duration of COVID-19 associated symptoms, hospitalization, and recovery time from the infection in Bangladesh.

**Methods:** A retrospective study was designed adopting the snowball sampling technique (*n* = 439). The association of gender, age, and comorbidity on COVID-19 associated complications was determined using chi-square and binary logistic regression analysis (*p* < 0.05).

**Result:** Fever, exhaustion, cough, loss of taste, sore throat, body ache, and hair-loss were prevalent among more than 50% of the participants and developed within fourth days in above 90% of the patients. Shortness of breath was significantly higher in males (χ2 = 5.671; OR 1.641). Significant comorbidity association on the shortness of breath (χ2 = 40.119; OR 2.564), vomiting (χ2 = 4.422; OR 1.018), and loss of speech (χ2 = 17.299; OR 3.430) was observed. Patients (>40 years) exerted higher association in shortness of breath (χ2 = 24.083; OR 2.901). Age and comorbidity were significantly associated with COVID-19 associated hospitalization (χ2 = 16.890 and χ2 = 23.638, respectively) and recovery time (χ2 = 12.870 and χ2 = 26.924, respectively).

**Conclusion:** The study suggests that the hospitalization rate increased for older (>40 years) and comorbid patients. Comorbid patients demonstrated higher susceptibility to have shortness of breath, vomiting, loss of speech, and confusion, whereas male patients showed significant increase in the prevalence of sore throat, loss of smell, and vomiting compared to female patients.

## Introduction

In late December 2019, a small number of inexplicable respiratory infections were recorded in Wuhan, Hubei province, China. It has clinical similarities to viral pneumonia and Prevention Research Centers, Centers for Disease Control (CDC) experts determined that pneumonia was caused by a novel β-coronavirus (1, 2). Afterward, WHO relabeled the name of this virus as severe acute respiratory syndrome coronavirus-2 (SARS-CoV-2) suggested by the Coronavirus Study Group of the International Committee on Taxonomy of Viruses (ICTV), and the disease was termed as coronavirus disease 2019 or simply COVID-19 (3). Although no animal source has been confirmed yet, due to the similarity in genomic features, it is postulated that bat or pangolin may work as a reservoir of SARS-CoV-2 (4). On Mar 11, 2020, WHO stated the COVID-19 outbreak as a global pandemic as reported cases reach 200,000 people, with over 8,000 people died due to the complications related to COVID-19 in over 160 countries (5, 6). The number of confirmed cases increases every day, exerting a wide variety of symptoms in the infected persons. SARS-CoV-2 has now spread to more than 213 countries worldwide.

Coronaviruses are a broad family of viruses that cause respiratory disorders, which diagnosis varies from the common cold to more severe disorders such as Middle East Respiratory Syndrome (MERS), Severe Acute Respiratory Syndrome (SARS), and COVID-19 (7). The symptoms of coronaviruses infections include cough, fever, diarrhea, chest pain, fatigue, body ache, sore throat, rhinorrhea, tachypnea, dyspnea (7, 8). The presence of glycoprotein spikes on the envelope of this RNA virus gives it a classic crown-like look under an electron microscope (9). This group of viruses is zoonotic, meaning they can be transmitted between animals and people (10–12). Coronaviruses are classified into four genera, one of which is α-/β-/γ-/δ-coronavirus. The α- and β-coronaviruses are both capable of infecting mammals, while γ- and δ-coronaviruses are more commonly found in birds (13). Six coronaviruses had long been described as human-susceptible viruses. Among them, α-coronavirus HCoV-229E and HCoV-NL63 bear low pathogenicity, and β-CoVs HCoV-HKU1 and HCoV-OC43 cause moderate respiratory symptoms, identical to a common cold. On the other hand, the other known β-CoVs, SARS-CoV and MERS-CoV could result in a severe and potentially fatal respiratory illness (14, 15). The disease is generally known to be transmitted between animals and humans through sneezing, coughing, touching or shaking hands, and contacting a surface or object and the incubation period ranges from 2 to 14 days (9, 14). The infection has been predicted by a loss of smell and taste in addition to other symptoms (16, 17). The prevalence of gastrointestinal symptoms before fever and difficulty in breathing was also reported in a previous study (18). COVID-19 has caused millions of people to be hospitalized around the world, with symptoms including fever, dry cough, exhaustion, diarrhea, breathing problem, headache, nausea, and vomiting (19). After 60 days, just 13% of people in Italy were completely free of COVID-19 symptoms, with 55% reporting three or more persistent symptoms and 32% reporting one or two residual symptoms (20). Similarly, COVID-19 patients who suffered from chronic symptoms exhibited a lower life expectancy after 2–3 weeks of infection in the United States (21).

COVID-19 associated symptoms and complications may vary from person to person. COVID-19 has a wide range of frequency and severity, which is believed to be influenced by various biological, cultural, and economic factors. Many of those who died had other advanced age, hypertension, diabetes, or cardiovascular disease that impaired their immune systems (14). According to previous clinical investigations, females are less likely to be infected and have lower cytokine production than previous clinical investigations (22). African and Asian races have a higher chance of acquiring COVID-19 than Caucasian people; Asians may also have a higher chance of hospitalization and mortality (23).

**Figure 1:**
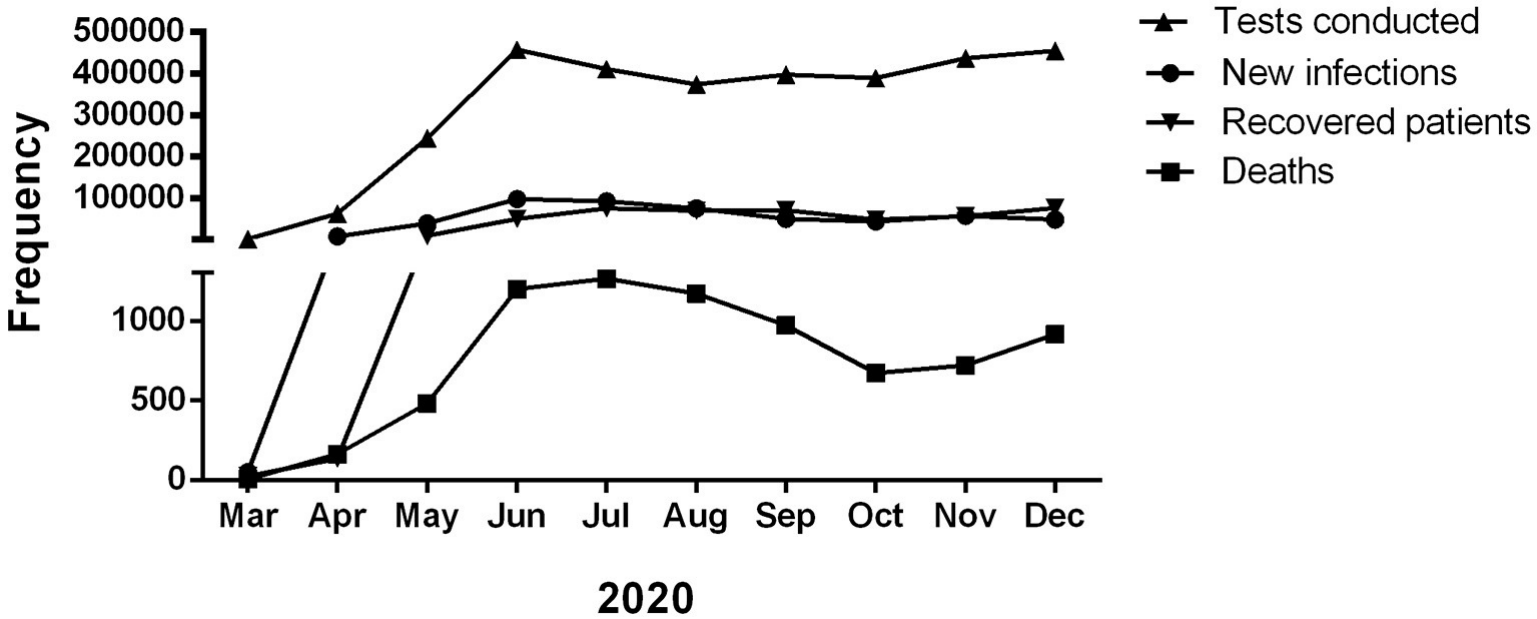
Month-wise total number of COVID-19 patients, death, recovered, and RT-PCR test conducted in Bangladesh in 2020 (Ref: DGHS, Bangladesh).

In Bangladesh, the first documented incidence was reported on Mar 08, 2020 (24). Infection rates remained low until the end of March, following which a significant surge in infected cases occurred that persisted till June 2020 (25, 26). The number of new COVID-19 infection cases, COVID-19-related deaths, recovery from COVID-19, and the number of Real-Time Reverse Transcription Polymerase Chain Reaction (RT-PCR) tests conducted in Bangladesh is illustrated in Figure 1. There has been very limited information regarding the COVID-19 symptoms and associated consequences among the patient of Bangladesh. In this study, we were focused on assessing the prevalence, onset, and duration of the severity of the symptoms and recovery time in the study participants. We also tried to determine the impact of different factors such as gender, age, and comorbidity on hospitalization, recovery time as well as the prevalence of the COVID-19 associated symptoms.

## Methods

### Study Design and Time

A cross-sectional study design using “Google Form” was adopted to assess the prevalence of COVID-19-related symptoms among the patients of Bangladesh during the outbreak of COVID-19. Data were collected from Sept 08, 2020, to Nov 08, 2020. At that time, it was not feasible to conduct community-based surveys due to the countrywide lockdown implemented by the government (27, 28). This self-reported retrospective study was conducted using a standardized questionnaire. Only those infected with coronavirus between Mar 08, 2020, and Aug 08, 2020, had their infection verified by RT-PCR test and then recovered from the condition- were included in the study.

### Sampling of the Study

The online link was circulated by the Exponential Non-Discriminative snowballing method (29, 30). An online-based advertisement campaign was designed to recruit participants for this online survey who suffered and eventually recovered from COVID-19 (31). Participants residing in Bangladesh were randomly selected, and none of them were related to any study. We covered the people who use social media platforms (e.g., Facebook and LinkedIn) to collect information for this study. People receiving the message were requested to participate in the survey and forward the link to their close contacts infected by this coronavirus. Participants whose age was 18 years old or above were allowed to participate in this study. A total of 453 complete responses were received in this survey who agreed to take part. Of these, six responses were also omitted due to incomplete data. In addition, answers from the participants having COVID-19 like symptoms but not performing the RT-PCR test- were also excluded. Finally, a total of 439 responses were considered for final data analysis.

### Study Tool Preparation

The survey consisted of 23 questions. Except for two unstructured, open-ended questions, all of the questions were closed-ended. The questionnaire has four domains. The first one collected the demographic details of the participants. The second domain was constructed to contain participants' infection date, RT-PCR test information (to ascertain the infection and recovery from the disease), treatment, hospitalization and recovery time, and other comorbidity histories. The questions of the third and fourth domains were designed to measure the onset and duration of the symptoms, respectively. A set of nineteen symptoms was included in the questionnaire to check the symptoms' prevalence, onset, and duration. In addition, participants were allowed to report any other symptoms that were not mentioned in the questionnaire through an open-ended question. The history of other chronic diseases was also recorded. The questions were constructed in English to understand the scientific terminologies better, whereas a Bengali (most participants' mother tongue) translation was also provided. A forward-backward translation process was followed for the finalization of the questionnaire.

### Data Management

The data from the “Google Form” was initially extracted in.xlsx format. Participants who disagreed with using their information, those who did not complete an RT-PCR test to confirm infection, and incomplete responses were then deleted from this study. After that, the data was converted to.sav format for further statistical analysis. Descriptive statistics (frequency, percentages, and means) were used to examine the participants' responses and demographic distribution. Participants were divided into different groups for further analysis based on gender (male-female), age (aging ≤40 years old and aging >40 years old), and comorbidity (other chronic disease(s)-no other disease(s) during the infection) based on other relevant conditions (32, 33). The Pearson's chi-squared test followed by binary logistic regression analysis was applied to determine the association of different predictors in the prevalence of the symptoms. Statistical significance was defined when *p* < 0.05. All statistical tests were performed using STATA 12.0 and IBM SPSS 23.0 version.

### Ethical Consideration

The current study was carried out following the Helsinki declaration changed in 2014 and the checklist for reporting internet e-surveys (CHERRIES) (34, 35). The study was reviewed and approved by The Jagannath University Research Cell, Dhaka, Bangladesh (File no.- JnURes-001/2020). The objectives, potential benefits, risks, and the confidentiality of given responses were communicated with participants before starting the online survey. Participants also received an option to provide their consent to use their provided information for further analysis in the study.

## Results

### Demographics

The final analysis consisted of 439 respondents where 69% (*n* = 303) were male, and 31% (*n* = 136) were female (Table 1). The participants' ages ranged from 18 years to above 60 years. About 42% (*n* = 183) participants aged from 18 to 30 years followed by 31% (*n* = 135) people aged from 31 to 40 years. Although respondents were from all divisions of Bangladesh, the highest response rate was from Barishal Division (49%, *n* = 215), whereas the lowest was from the Rangpur division (0.7%, *n* = 3). The division wise participant information is illustrated in Supplementary Figure 1.

**Table 1:** Demographic distribution of the participants, Bangladesh, 2020.

**Variables**	**Total (%)**	**Male (%)**	**Female (%)**
Participants	439 (100%)	303 (69%)	136 (31%)
**Age**			
16–30 Yrs	183 (41.7%)	112 (37%)	71 (52.2%)
31–40 Yrs	135 (30.8%)	101 (33.3%)	34 (25%)
41–50 Yrs	66 (15%)	50 (16.5%)	16 (11.8%)
51–60 Yr	40 (9.1%)	31 (10.2%)	9 (6.6%)
Above 60 Yrs	15 (3.4%)	9 (3%)	6 (4.4%)
**Divisional location**			
Barishal	215 (49%)	146 (48.2%)	69 (50.7%)
Chittagong	17 (3.9%)	10 (3.3%)	7 (5.1%)
Dhaka	103 (23.5%)	71 (23.4%)	32 (23.5%)
Khulna	37 (8.4%)	22 (7.3%)	15 (11%)
Mymensingh	3 (0.7%)	2 (0.7%)	1 (0.7%)
Rajshahi	7 (1.6%)	4 (1.3%)	3 (2.2%)
Rangpur	3 (0.7%)	2 (0.7%)	1 (0.7%)
Sylhet	54 (12.3%)	46 (15.2%)	8 (5.9%)
**Occupation**			
Govt. job	111 (25.3%)	89 (29.4%)	22 (16.2%)
Housewife	58 (13.2%)	0 (0.0%)	50 (36.8%)
Private job	122 (27.8%)	105 (34.7%)	17 (12.5%)
Self-employed	53 (12.1%)	49 (16.2%)	4 (2.9%)
Student	87 (19.8%)	48 (15.8%)	39 (28.7%)
Unemployed	8 (1.8%)	12 (3.9%)	4 (2.9%)
**Monthly income**			
Undisclosed	12 (2.7%)	5 (1.7%)	7 (5.1%)
< BDT 10,000	130 (29.6%)	51 (16.9%)	77 (56.6%)
BDT 10,000–20,000	75 (17.1%)	63 (20.9%)	12 (8.8%)
BDT 21,000–40,000	134 (30.5%)	107 (35.5%)	27 (19.8%)
BDT 41,000–60,000	53 (12.1%)	42 (13.9%)	11 (8%)
More than BDT 60,000	35 (8%)	33 (11%)	2 (1.5%)
**Month of infection**			
March-April, 2020	8 (1.8%)	6 (2%)	2 (1.5%)
May-June, 2020	124 (28.2%)	92 (30.4%)	32 (23.5%)
July-August, 2020	160 (36.4%)	105 (34.7%)	55 (40.4%)
September-October, 2020	147 (33.5%)	100 (33%)	47 (34.5%)

Out of all the participants, 44.5% were students, and most non-student participants had a baccalaureate degree. There was a larger response among those with higher secondary education and graduates. The majority of the respondents (more than 70%) work in the private sector, whereas some were government job holders (25.28%) healthcare professionals and mostly worked or did Private Job holder or business (39.86) in a crowded place, 19.81% were students, and 18 % of the respondents reported that a relative, colleagues or a neighbor had been diagnosed with COVID-19.

One-third (29.6%, *n* = 130) of respondents' monthly income was below 10,000 BDT (117.81 USD), whereas another one-third (30.5%, *n* = 134) of respondent's monthly income was between 21,000 BDT to 40,000 BDT (247.41 USD to 471.27 USD). The exchange rate was considered 1 USD = 84.8775 (average of day's lowest and day's highest, data obtained from www.bb.org.bd/econdata/exchangerate.php on June 25th, 2021). Monthly income of above 60,000 BDT (706.9 USD) consist of 8% (*n* = 35), where 2.7% (*n* = 12) did not disclose the information (Table 1).

### Comorbidity

The prevalence and severity of the COVID-19 associated symptoms tend to be impacted by comorbidities. Therefore, participants were asked to report any chronic disease(s) they had been suffering from. Among 439 participants, 26.19% of participants (a total of 115 participants, among them 76 male and 39 female) reported that they had been suffering from different chronic diseases(s) or comorbidities (single or multiple chronic diseases) before being infected by the coronavirus, such as asthma, chronic obstructive pulmonary disease (COPD), diabetes, hypertension, stroke, heart attack, other heart diseases, kidney disease, allergy, arthritis, liver disease, and obesity. The self-reported comorbidities were later categorized as Lung disease (asthma, COPD, and other respiratory problems), diabetes, cardiac disease (hypertension, stroke, heart attack, and other heart diseases), and miscellaneous (kidney disease, allergy, rhinitis, liver disease, and obesity). 18.67% of total participants (*n* = 82) had cardiac disease, 6.83% (*n* = 30) had diabetes, 5% (*n* = 22) had lung disease, and the rest of the comorbid participants (2.96%, *n* = 13) were suffering from miscellaneous chronic conditions.

### COVID-19 Complications Associated With Hospitalization

The obtained result found that COVID-19-related complications led to the admission of 13% of participants to the hospital (Table 2). Comorbid patients were more prone to be hospitalized due to COVID-19 associated complications. Significant association in gender (χ2 = 12.870, *p* = 0.000) and comorbidity (χ2 = 26.924, *p* = 0.000) was also found in hospitalization due to COVID-19 associated complications.

**Table 2:** Hospitalization due to COVID-19 associated complications, Bangladesh, 2020.

**Variable**	**Group**	**Admitted into hospital**	**Home quarantined**	**χ^2^** **(***p***-value)**
Total participants	*N* = 439	57 (12.99%)	382 (87.01%)	
Gender	Male (*n* = 303)	45 (14.85%)	258 (85.15%)	3.019 (0.082)
	Female (*n* = 136)	12 (8.82%)	124 (91.18%)	
Age	≤40 Yrs (*n* = 318)	30 (9.43%)	288 (90.57%)	12.870 (0.000)
	>40 Yrs (*n* = 121)	27 (22.31%)	94 (77.69%)	
Comorbidity	Yes (*n* = 115)	31 (26.95%)	84 (73.05%)	26.924 (0.000)
	No (*n* = 324)	26 (8.02%)	298 (91.98%)	

*Values are expressed as frequency and % response of the final respondents. The percentage was calculated considering the samples in the individual groups. Pearson's chi-squared test was applied to observe the association of gender, age, and the previous history of comorbidity in the incidence of hospitalization. P < 0.05 was considered statistically significant*.

### Prevalence of COVID-19 Associated Symptoms

Table 3 shows the prevalence of COVID-19-related symptoms. Table 4 depicts how male gender, older age (>40 years), and comorbidity were associated (odds ratio) with the prevalence of these symptoms among the participants. Fever (93.60%), exhaustion (88.80%), and cough (70.80%) were the most prevalent symptoms reported by the participants. Loss of taste, sore throat, body ache, and hair loss were common among more than 50%. Nearly half of the respondents (49.70%) complained about the loss of smell due to the infection. Significant gender association was observed in sore throat (χ2 = 4.210; *p* = 0.040), loss of smell (χ2 = 5.671; *p* = 0.017), and vomiting (χ2 = 4.724; *p* = 0.030). The findings revealed that gender and comorbidities also had an impact on the characteristics of COVID-19-related symptoms. Older adults (>40 years) were significantly associated with shortness of breath (χ2 = 24.083; *p* = 0.000) and hair loss (χ2 = 38.887; *p* = 0.000). A significant difference was observed in the group based on comorbidity in shortness of breath (χ2 = 40.119; *p* = 0.000), vomiting (χ2 = 4.442; *p* = 0.035), confusion (χ2 = 15.558; *p* = 0.000), Loss of speech (χ2 = 17.299; *p* = 0.000) and hair loss (χ2 = 20.212; *p* = 0.000). Male patients showed 1.641 times higher odds (95% CI 1.090–2.472) of having the symptom of loss of smell than the female patients. Patients over 40 years old were significantly associated with shortness of breath [OR = 2.901 (95% CI 1.881–4.476)]. According to the findings, comorbidity was also found to be substantially linked with the prevalence of shortness of breath [OR = 4.042; (95% CI 2.564–6.374)], vomiting [OR = 1.689; (95% CI 1.018–2.802)], loss of speech [OR = 3.430 (1.944–6.053)], and Confusion [OR = 2.746 (95% CI 1.686–4.476)].

**Table 3:** Prevalence of the Covid-19 associated symptoms among the participants, Bangladesh, 2020.

**Response**	**Total frequency (%)**	**Male frequency (%)**	**Female frequency (%)**	**χ^2^** **(***p***-Value)**	**≤40 Yrs frequency (%)**	**>40 Yrs frequency (%)**	**χ^2^** **(***p***-Value)**	**No Comorbidity**	**Comorbidity**	**χ^2^** **(***p***-Value)**
Fever	411 (93.6%)	285 (94.1%)	126 (92.6%)	0.314 (0.576)	296 (93.2%)	115 (95.0%)	0.564 (.453)	306 (94.2%)	105 (92.1%)	0.593 (0.441)
Runny Nose	95 (21.6%)	6 (19%)	35 (25.7%)	1.949 (0.163)	70 (22.0%)	25 (20.7%)	0.094 (.759)	67 (20.6%)	28 (24.6%)	0.775 (0.379)
Cough	311 (70.8%)	207 (68.3%)	104 (76.5%)	3.021 (0.082)	220 (69.2%)	91 (75.2%)	1.540 (0.215)	224 (68.9%)	87 (76.3%)	2.233 (0.135)
Sore throat	279 (63.6%)	183 (60.40%)	96 (70.6%)	4.21 (0.04)	208 (65.4%)	71 (58.7%)	1.714 (0.190)	212 (65.2%)	67 (58.8%)	1.520 (0.218)
Shortness of breath	193 (44%)	128 (42.2%)	65 (47.8%)	1.174 (0.279)	117 (36.8%)	76 (62.8%)	24.083 (.000)	114 (35.1%)	79 (69.3%)	40.119 (0.000)
Body ache	275 (62.6%)	196 (64.7%)	79 (58.1%)	1.746 (0.186)	199 (62.6%)	76 (62.8%)	0.002 (.964)	199 (61.2%)	76 (66.7%)	1.066 (0.302)
Diarrhea	135 (30.8%)	95 (31.4%)	40 (29.40%)	0.166 (0.684)	93 (29.2%)	42 (34.7%)	1.229 (0.268)	92 (28.3%)	43 (37.7%)	3.511 (0.061)
Loss of taste	281 (64%)	201 (66.3%)	80 (58.8%)	2.3 (0.129)	204 (64.2%)	77 (63.6%)	0.912 (.502)	208 (64.0%)	73 (64.0%)	0.000 (0.995)
Loss of smell	218 (49.7%)	162 (53.5%)	56 (41.2%)	5.671 (0.017)	156 (49.1%)	62 (51.2%)	0.167 (0.683)	162 (49.8%)	56 (49.1%)	0.018 (0.894)
Vomiting	86 (19.6%)	51 (16.8%)	35 (25.7%)	4.724 (0.03)	60 (18.9%)	26 (21.5%)	0.382 (0.537)	56 (17.2%)	30 (26.3%)	4.422 (0.035)
Headache	154 (35.1%)	101 (33.3%)	53 (39%)	1.31 (0.252)	117 (36.8%)	37 (30.6%)	1.486 (0.223)	112 (34.5%)	42 (36.8%)	0.210 (0.647)
Digestive problem	65 (14.8%)	41 (13.5%)	24 (17.6%)	1.261 (0.262)	48 (15.1%)	17 (14.0%)	0.076 (0.783)	47 (14.5%)	18 (15.8%)	0.118 (0.731)
Lack of appetite	173 (39.4%)	114 (37.6%)	59 (43.4%)	1.304 (0.254)	120 (37.7%)	53 (43.8%)	1.351 (0.245)	123 (37.8%)	50 (43.9%)	1.278 (0.258)
Rashes	44 (10%)	31 (10.20%)	13 (9.60%)	0.047 (0.828)	33 (10.4%)	11 (9.1%)	0.161 (0.688)	32 (9.8%)	12 (10.5%)	0.043 (0.835)
Blistering	10 (2.3%)	7 (2.3%)	3 (2.2%)	0.005 (0.946)	7 (2.2%)	3 (2.5%)	0.030 (0.861)	5 (1.5%)	5 (4.4%)	3.074 (0.080)
Confusion	90 (20.5%)	62 (20.50%)	28 (20.6%)	0.001 (0.976)	62 (19.5%)	28 (23.1%)	0.714 (0.398)	52 (16.0%)	38 (33.3%)	15.558 (0.000)
Loss of speech	58 (13.2%)	43 (14.2%)	15 (11%)	0.819 (0.366)	39 (12.3%)	19 (15.7%)	0.904 (0.342)	30 (9.2%)	28 (24.6%)	17.299 (0.000)
Exhaustion	390 (88.8%)	268 (88.4%)	122 (89.7%)	0.15 (0.699)	281 (88.4%)	109 (90.1%)	0.261 (0.610)	286 (88.0%)	104 (91.2%)	0.887 (0.346)
Hair loss	222 (50.6%)	159 (52.5%)	63 (46.3%)	1.421 (0.233)	190 (59.7%)	32 (26.4%)	38.887 (0.000)	185 (56.9%)	37 (32.5%)	20.212 (0.000)

*Values are expressed as frequency and % response of the final respondents. Total participants n=439; Male n= 303, Female n = 136; Age ≤40 years old, n = 318, Age >40 years old, n = 121. The percentage was calculated within the group. Pearson's chi-squared test was applied to observe the association of gender (male and female), age (≤40 years old and >40 years old patients), and comorbidity in the prevalence of Covid-19 associated symptoms. P < 0.05 was considered statistically significant*.

**Table 4:** Odds Ratios of Covid-19 associated symptoms, Bangladesh, 2020.

**Symptom**	**OR [CI] Male**	**OR [CI] >40 years old**	**OR [CI] Comorbidity**
Fever	1.257 [0.564–2.799]	1.425 [0.563–3.603]	0.734 [0.322–1.671]
Runny Nose	0.713 [0.442–1.148]	0.923 [0.552–1.542]	1.318 [0.799–2.174]
Cough	0.663 [0.417–1.055]	1.351 [0.839–2.176]	1.387 [0.853–2.257]
Sore Throat	0.635 [0.411–0.982]^*^	0.751 [0.489–1.154]	0.774 [0.501–1.199]
Shortness of Breath	0.799 [0.532–1.200]	2.901 [1.881–4.476]^***^	4.042 [2.564–6.374]^***^
Body Ache	1.322 [0.873–2.000]	1.010 [0.655–1.557]	1.224 [0.783–1.912]
Diarrhea	1.096 [0.705–1.705]	1.286 [0.824–2.008]	1.506 [0.962–2.358]
Loss of Taste	1.379 [0.910–2.092]	0.978 [0.633–1.512]	0.969 [0.623–1.509]
Loss of Smell	1.641 [1.090–2.472]^*^	1.091 [0.718–1.659]	0.949 [0.620–1.453]
Vomiting	0.584 [0.358–0.952]^*^	1.177 [0.702–1.973]	1.689 [1.018–2.802]^*^
Headache	0.783 [0.515–1.191]	0.757 [0.483–1.185]	1.146 [0.737–1.783]
Digestive Problem	0.730 [0.421–1.266]	0.919 [0.506–1.671]	1.196 [0.668–2.142]
Loss of appetite	0.787 [0.522–1.187]	1.286 [0.841–1.967]	1.257 [0.816–1.935]
Rashes	1.078 [0.545–2.132]	0.864 [0.422–1.769]	1.063 [0.528–2.142]
Blistering	1.048 [0.267–4.117]	1.130 [0.287–4.441]	2.900 [0.824–10.207]
Loss of Speech	1.334 [0.713–2.495]	1.333 [0.736–2.412]	3.430 [1.944–6.053]^***^
Confusion	0.992 [0.601–1.637]	1.243 [0.750–2.061]	2.746 [1.686–4.476]^***^
Extreme Exhaustion	0.879 [0.456–1.693]	1.196 [0.601–2.379]	1.256 [0.619–2.549]
Hair Loss	1.279 [0.853–1.919]	0.242 [0.153–0.384]^***^	0.375 [0.240–0.587]

*OR = odds ratio; [CI] = 95% confidence interval. OR > 1 means greater odds of association with the exposure and outcome; OR = 1 means there is no association between exposure and outcome; OR < 1 means there is a lower odds of association between the exposure and outcome. Logistic binary regression analysis was applied to determine the correlation of gender (male), age (>40 years old) and comorbidity (previous history of other chronic diseases) on the prevalence of the Covid-19 associated symptoms*.

* *p < 0.05*,

*** *p < 0.001*.

### Onset and Duration of COVID-19 Associated Symptoms

The onset and duration of COVID-19 related symptoms are described in Table 5 and Table 6, respectively. In brief, the most common symptom in COVID-19 patients was fever (93.6%, *n* = 411), which lasted for 1–4 days for most of the patients (77.4%, *n* = 340). The second most common symptom was exhaustion, reported in 89.8% (*n* = 390) patients and lasted for 11–14 days for 50% of the patients who have the symptom. Other accompanying symptoms were cough, body ache, loss of taste, loss of smell, lack of appetite, and headache, whereas more than 100 patients were affected by those symptoms. Out of all the symptoms we reported, exhaustion, sore throat, shortness of breath, body ache, vomiting, headache, digestive problems, lack of appetite, blistering, Confusion, and/or loss of speech were developed in some patients 14th days of infection. Interestingly, the onset of blistering was after 14th days for 20% of patients (*n* = 2). However, all the symptoms persisted in some patients for more than 14 days, whereas 33% (*n* = 127) exhibited exhaustion for more than 14 days. Similarly, Confusion occurred for more than 14 days in 27% of patients (*n* = 22).

**Table 5:** Onset of COVID-19-associated symptoms in the patients, Bangladesh, 2020.

**Symptom**	**Group**	**1st−4th day**	**5th−10th day**	**11th−14th day**	**After 14th day**
Fever	Male (*n* = 285)	277 (97.2%)	8 (2.8%)	0 (0.0%)	0 (0.0%)
	Female (*n* = 126)	118 (93.6%)	7 (5.6%)	1 (0.8%)	0 (0.0%)
	**Total**	**395 (96.1%)**	**15 (3.6%)**	**1 (0.2%)**	**0 (0.0%)**
Exhaustion	Male (*n* = 268)	243 (90.7%)	18 (6.7%)	4 (1.5%)	3 (1.1%)
	Female (*n* = 122)	104 (85.2%)	10 (8.2%)	5 (4.1%)	3 (2.5%)
	**Total**	**347 (89.0%)**	**28 (7.2%)**	**9 (2.3%)**	**6 (1.5%)**
Runny nose	Male (*n* = 60)	49 (81.7%)	11(18.3%)	0 (0.0%)	0 (0.0%)
	Female (*n* = 35)	28 (80.0%)	5(14.3%)	2 (5.7%)	0 (0.0%)
	**Total**	**77 (81.0%)**	**16 (16.8%)**	**2 (2.1%)**	**0 (0.0%)**
Cough	Male (*n* = 207)	183 (88.4%)	20 (9.7%)	4 (1.9%)	0 (0.0%)
	Female (*n* = 104)	91 (87.5%)	10 (9.6%)	3 (2.9%)	0 (0.0%)
	**Total**	**274 (88.1%)**	**30 (9.6%)**	**7 (2.2%)**	**0 (0.0%)**
Sore throat	Male (*n* = 183)	165 (90.2%)	16 (8.7%)	1 (0.5%)	1 (0.5%)
	Female (*n* = 96)	86 (89.6%)	9 (9.4%)	0 (0.0%)	1 (1.0%)
	**Total**	**251 (90.0%)**	**25 (9.0%)**	**1 (0.4%)**	**2 (0.7%)**
Shortness of breath	Male (*n* = 128)	44 (34.4%)	78 (60.9%)	3 (2.3%)	3 (2.3%)
	Female (*n* = 65)	22 (33.8%)	37 (56.9%)	4 (6.1%)	2 (3.1%)
	**Total**	**66 (34.2%)**	**115 (59.6%)**	**7 (3.6%)**	**5 (2.6%)**
Body ache	Male (*n* = 196)	180 (91.8%)	13 (6.6%)	1 (0.5%)	2 (1.0%)
	Female (*n* = 79)	73 (92.4%)	6 (7.6%)	0 (0.0%)	0 (0.0%)
	**Total**	**253 (92.0%)**	**19 (6.9%)**	**1 (0.4%)**	**2 (0.7%)**
Diarrhea	Male (*n* = 95)	76 (80.0%)	15 (15.8%)	4 (4.2%)	0 (0.0%)
	Female (*n* = 40)	32 (80.0%)	6 (15.0%)	2 (5.0%)	0 (0.0%)
	**Total**	**108 (80.0%)**	**21 (15.6%)**	**6 (4.4%)**	**0 (0.0%)**
Loss of taste	Male (*n* = 201)	71 (35.3%)	128 (63.7%)	2 (1.0%)	0 (0.0%)
	Female (*n* = 80)	34 (42.5%)	45 (56.2%)	1 (1.2%)	0 (0.0%)
	**Total**	**105 (37.4%)**	**173 (61.6%)**	**3 (1.1%)**	**0 (0.0%)**
Loss of smell	Male (*n* = 162)	67 (41.4%)	89 (54.9%)	6 (3.7%)	0 (0.0%)
	Female (*n* = 56)	18 (32.1%)	35 (62.5%)	3 (5.4%)	0 (0.0%)
	**Total**	**85 (39.0%)**	**124 (56.9%)**	**9 (4.1%)**	**0 (0.0%)**
Vomiting	Male (*n* = 51)	35 (68.6%)	14 (27.4%)	1 (2.0%)	1 (2.0%)
	Female (*n* = 35)	27 (77.1%)	5 (14.3%)	1 (2.9%)	2 (5.7%)
	**Total**	**62 (72.1%)**	**19 (22.1%)**	**2 (2.3%)**	**3 (3.5%)**
Headache	Male (*n* = 101)	89 (88.1%)	11 (10.9%)	0 (0.0%)	1 (1.0%)
	Female (*n* = 53)	48 (90.6%)	3 (5.7%)	0 (0.0%)	2 (3.8%)
	**Total**	**137 (89.0%)**	**14 (9.1%)**	**0 (0.0%)**	**3 (1.9%)**
Digestive problems	Male (*n* = 41)	26 (63.4%)	12 (29.3%)	1 (2.4%)	2 (4.9%)
	Female (*n* = 24)	16 (66.7%)	7 (29.2%)	1 (4.2%)	0 (0.0%)
	**Total**	**42 (64.6%)**	**19 (29.2%)**	**2 (3.1%)**	**2 (3.1%)**
Lack of appetite	Male (*n* = 114)	104 (91.2%)	9 (7.9%)	0 (0.0%)	1 (0.9%)
	Female (*n* = 59)	51 (86.4%)	6 (10.2%)	2 (3.4%)	0 (0.0%)
	**Total**	**155 (89.6%)**	**15 (8.7%)**	**2 (1.2%)**	**1 (0.6%)**
Rashes	Male (*n* = 31)	17 (54.8%)	12 (38.7%)	2 (6.4%)	0 (0.0%)
	Female (*n* = 13)	9 (69.2%)	1 (7.7%)	3 (23.1%)	0 (0.0%)
	**Total**	**26 (59.1%)**	**13 (29.5%)**	**5 (11.4%)**	**0 (0.0%)**
Blistering	Male (*n* = 7)	4 (57.1%)	1 (14.3%)	0 (0.0%)	2 (28.6%)
	Female (*n* = 3)	2 (66.7%)	1 (33.3%)	0 (0.0%)	0 (0.0%)
	**Total**	**6 (60.0%)**	**2 (20.0%)**	**0 (0.0%)**	**2 (20.0%)**
Confusion	Male (*n* = 62)	23 (37.1%)	21 (33.9%)	15(24.2%)	3 (4.8%)
	Female (*n* = 28)	5 (17.9%)	10 (35.7%)	9 (32.1%)	4 (14.3%)
	**Total**	**28 (31.1%)**	**31 (34.4%)**	**24 (26.7%)**	**7 (7.8%)**
Loss of speech	Male (*n* = 43)	18 (41.9%)	16 (37.2%)	8 (18.6%)	1 (2.3%)
	Female (*n* = 15)	4 (26.7%)	1 (6.7%)	2 (13.3%)	8 (53.3%)
	**Total**	**22 (37.9%)**	**24 (41.4%)**	**10 (17.2%)**	**2 (3.4%)**

*Values are expressed as frequency and % response of the final respondents. The percentage was calculated considering the samples in the individual groups*.

**Table 6:** Duration of the COVID-19-associated symptoms of the patients, Bangladesh, 2020.

**Symptoms**	**Group**	**1–4 days**	**5–10 days**	**11–14 days**	**>14 days**
Fever	Male	241 (85.2%)	34 (12.0%)	3 (1.1%)	5 (1.8%)
	Female	99 (79.8%)	23 (18.5%)	0 (0.0%)	2 (1.6%)
	**Total**	**340 (83.5%)**	**57 (14.0%)**	**3 (0.7%)**	**7 (1.7%)**
Exhaustion	Male	17 (6.6%)	23 (8.9%)	125 (48.6%)	92 (35.8%)
	Female	10 (8.4%)	11 (9.2%)	63 (52.9%)	35 (29.4%)
	**Total**	**27 (7.2%)**	**34 (9.0%)**	**188 (50.0%)**	**127 (33.8%)**
Runny nose	Male	49 (74.2%)	13 (19.7%)	2 (3.0%)	2 (3.0%)
	Female	21 (63.6%)	5 (15.1%)	3 (9.1%)	4 (12.1%)
	**Total**	**70 (70.7%)**	**18 (18.2%)**	**5 (5.0%)**	**6 (6.1%)**
Cough	Male	141 (68.8%)	44 (21.5%)	11 (5.4%)	9 (4.4%)
	Female	74 (74.0%)	12 (12.0%)	7 (7.0%)	7 (7.0%)
	**Total**	**215 (70.5%)**	**56 (18.4%)**	**18 (5.9%)**	**16 (5.2%)**
Sore throat	Male	168 (92.8%)	9 (5.0%)	2 (1.1%)	2 (1.1%)
	Female	84 (86.6%)	10 (10.3%)	1 (1.0%)	2 (2.1%)
	**Total**	**252 (90.6%)**	**19 (6.8%)**	**3 (1.1%)**	**4 (1.4%)**
Shortness of breath	Male	20 (17.5%)	39 (34.2%)	50 (43.9%)	11 (9.6%)
	Female	14 (22.9%)	29 (47.5%)	12 (19.7%)	6 (9.8%)
	**Total**	**34 (18.8%)**	**68 (37.6%)**	**62 (34.2%)**	**17 (9.4%)**
Body ache	Male	105 (55.3%)	68 (35.8%)	10 (5.3%)	7 (3.7%)
	Female	38 (49.35%)	28 (36.4%)	7 (9.1%)	4 (5.2%)
	**Total**	**143 (53.6%)**	**96 (36.0%)**	**17 (6.4%)**	**11 (4.1%)**
Diarrhea	Male	81 (88.0%)	7 (7.6%)	3 (3.3%)	1 (1.1%)
	Female	28 (80.0%)	4 (11.4%)	3 (8.6%)	0 (0.0%)
	**Total**	**109 (85.8%)**	**11 (8.7%)**	**6 (4.7%)**	**1 (0.8%)**
Loss of taste	Male	12 (6.0%)	31 (15.4%)	140 (69.6%)	18 (9.0%)
	Female	8 (10.3%)	23 (29.5%)	39 (50.0%)	8 (10.3%)
	**Total**	**20 (7.2%)**	**54 (19.3%)**	**179 (64.2%)**	**26 (9.3%)**
Loss of smell	Male	15 (9.2%)	30 (18.6%)	99 (61.5%)	17 (10.6%)
	Female	5 (8.93%)	21 (37.50%)	23 (41.1%)	7 (12.5%)
	**Total**	**20 (9.2%)**	**51 (23.5%)**	**122 (56.2%)**	**24 (11.1%)**
Vomiting	Male	28 (54.9%)	21 (41.2%)	2 (3.9%)	0 (0.0%)
	Female	16 (51.6%)	9 (29.0%)	3 (9.7%)	3 (9.7%)
	**Total**	**44 (53.7%)**	**30 (36.6%)**	**5 (6.1%)**	**3 (3.7%)**
Headache	Male	82 (71.9%)	16 (14.0%)	7 (6.1%)	9 (7.9%)
	Female	40 (71.4%)	6 (10.7%)	7 (12.5%)	3 (5.4%)
	**Total**	**122 (71.8%)**	**22 (13.0%)**	**14 (8.2%)**	**12 (7.1%)**
Digestive problems	Male	10 (27.8%)	22 (61.1%)	1 (2.8%)	3 (8.3%)
	Female	10 (47.6%)	6 (28.6%)	2 (9.5%)	2 (9.5%)
	**Total**	**20 (35.1%)**	**28 (49.1%)**	**4 (7.0%)**	**5 (8.8%)**
Lack of appetite	Male	28 (25.2%)	73 (65.8%)	5 (4.5%)	5 (4.5%)
	Female	15 (25.4%)	35 (59.3%)	7 (11.9%)	2 (3.4%)
	**Total**	**43 (25.3%)**	**108 (63.5%)**	**12 (7.1%)**	**7 (4.1%)**
Rashes	Male	23 (74.2%)	7 (22.6%)	0 (0.0%)	1 (3.2%)
	Female	9 (81.8%)	0 (0.0%)	0 (0.0%)	2 (18.2%)
	**Total**	**32 (76.2%)**	**7 (16.7%)**	**0 (0.0%)**	**3 (7.1%)**
Blistering	Male	5 (71.4%)	1 (14.3%)	0 (0.0%)	1 (33.3%)
	Female	1 (33.3%)	1 (33.3%)	0 (0.0%)	1 (33.3%)
	**Total**	**6 (60.0%)**	**2 (20.0%)**	**0 (0.0%)**	**2 (20.0%)**
Confusion	Male	24 (42.1%)	14 (24.6%)	4 (7.0%)	15 (26.3%)
	Female	9 (36.0%)	3 (12.0%)	6 (24.0%)	7 (28.0%)
	**Total**	**33 (40.2%)**	**17 (20.7%)**	**10 (12.2%)**	**22 (26.8%)**
Loss of speech	Male	17 (41.5%)	12 (29.3%)	4 (9.8%)	8 (19.5%)
	Female	8 (50.0%)	3 (18.7%)	3 (18.7%)	2 (12.5%)
	**Total**	**25 (43.9%)**	**15 (26.3%)**	**7 (12.3%)**	**10 (17.5%)**

*Values are expressed as frequency and % response of the final respondents). The percentage was calculated considering the samples in the individual groups*.

### Time Required for the Recovery From the COVID-19 Associated Symptoms

In various patients, the time it takes to get a coronavirus infection negative by RT-PCR test may differ from the time it takes to recover from all symptoms. The duration required to get rid of all the COVID-19 associated symptoms was also assessed in this study and illustrated in Table 7. More than 53% of patients were recovered from all COVID-19 related symptoms within 8 to 14 days. However, most patients older than 40 years (45.5%) required 15–21 days to recover from the symptoms. Significant differences in the groups of age (χ2 = 16.890; *p* = 0.005) and comorbidity (χ2 = 23.638; *p* = 0.040) was observed in time required for the complete symptomatic recovery.

**Table 7:** Days required for symptomatic recovery in both male and female patients, Bangladesh, 2020.

**Variable**	**Group**	**1–7 days**	**8–14 days**	**15–21 days**	**22–28 days**	**More than 28 days**	**χ^**2**^ (*p*-value)**
Total	*N* = 439	16 (3.6%)	233 (53.1%)	136 (31%)	7 (1.6%)	47 (10.7%)	–
Gender	Male (*n* = 303)	9 (3.0%)	152 (50.2%)	106 (35.0%)	3 (1.0%)	33 10.90%	10.157 (0.071)
	Female (*n* = 136)	7 (5.1%)	81 (59.6%)	30 (22.1%)	4 (2.9%)	14 (10.3%)	
Age	≤40 Yrs (*n* = 318)	12 (3.8%)	183 (57.5%)	81 (25.5%)	5 (1.6%)	37 (11.6%)	16.890 (0.005)
	>40 Yrs (*n* = 121)	4 (3.3%)	50 (41.3%)	55 (45.5%)	2 (1.7%)	10 (8.3%)	
Comorbidity	Yes (*n* = 115)	4 (3.5%)	41 (36.0%)	51 (44.7%)	4 (3.5%)	14 (12.3%)	23.638 (0.000)
	No (*n* = 324)	12 (3.7%)	192 (59.1%)	85 (26.2%)	3 (0.9%)	33 (10.2%)	

*Values are expressed as frequency and % response of the final respondents. The percentage was calculated considering the samples in the individual groups. Pearson's chi-squared test was applied to observe the association of the gender, age, and comorbidity in the complete recovery of all COVID-19 associated symptoms. P < 0.05 was considered statistically significant*.

## Discussion

In the last 20 years, several respiratory-related viral diseases such as SARS-CoV, H1N1 influenza, and MERS-CoV epidemics outbroke (36). Although previous coronaviruses, SARS-CoV and MERS-CoV, exhibited a high death rate, 9.6, and 35%, respectively, SARS-CoV-2 was declared as pandemic because of its high contagiousness and global spreading (36).

This study was conducted on 439 people in Bangladesh who recovered from COVID-19 at least 1 month before participating. Conforming to the first COVID-19 patient detection in Bangladesh, the survey includes the patients who got infected between March 2020 to September 2020. Our study found that most participants got infected from May-June, which matches the data obtained from the Directorate General of Health Services (DGHS) (Figure 1). Only 8 (1.8%) respondents got infected between March and April, suggesting that the virus took about 2 months to widespread. Most patients were between the ages above 40 years, and the male patient rate was 74.38%.

When we try to assess the relationship between hospitalization rate and predictors such as gender, age, or comorbidity, we found elderly patients had a significantly high hospitalization rate (22.31 vs. 9.43%), and 26.95% of patients with comorbidity were hospitalized compared to the patients (8.02%) who were hospitalized without any comorbid condition. The data suggest that age and comorbid conditions play a significant role in the severity of COVID-19. A recent study also found that the elder patients group (median age 69) had a long time of illness onset to hospitalization compared to the younger patients group (median age 40) (37). The study conducted in rural US patients found that hospitalized patients had a higher comorbidity burden (38). However, one of the major drawbacks of this cross-sectional study is determining the disease conditions. Since most were treated at home, we excluded some symptoms such as pneumonia because of a complicated diagnosis. Another limitation could include the lack of mortality data since we did not include deceased patients.

Next, we conducted a chi-square test to study the role of gender, age, and comorbidity in the prevalence of the Covid-19 associated symptoms. Out of all the symptoms we have tested, we found gender is a risk factor for sore throat and loss of smell, whereas aging and comorbidity could be a risk factors for shortness of breath and hair loss. Furthermore, we found a difference in symptomatic prevalence for vomiting, confusion, and loss of speech between the comorbid patients and the patients without any second disease. Further, odd ratios were calculated to assess the high risk of certain groups in the gender, age, and comorbidity predictors. Male patients showed a high-risk factor for the loss of smell symptom, whereas elderly patients had higher risk factors in developing shortness of breath than ≤40 years of age. Four symptoms such as shortness of breath, vomiting, loss of speech, confusion demonstrated high prevalence in comorbid patients.

One plausible reason for the gender differences could be due to hormonal differences. Female has shown higher incidence rate than male; however, female age between 20 s and 70 s exhibited lower incidence rate than similar age of male. The study suggests that estrogen may play a protective role with the other susceptible factors by inhibiting the entry of the SARS-CoV-2 virus into the host cells (39). Another important susceptible factor is age. A study conducted in 5,484 case contacts found that only 18.1% of participants developed symptoms below age 20, whereas 64.6% of participants demonstrated symptoms above age 80 (40). Chronic illnesses including hypertension, respiratory disease, kidney disease, diabetes, and cancer are considered underlying conditions that put people at greater risk with COVID-19. Hence we have included comorbid conditions (such as lung disease, cardiac disease, diabetes, and miscellaneous) to find their impacts on the symptoms. The study found that patients with comorbid disease/diseases had increased hospitalization rates and longer extended recovery periods. A retrospective study also described that aging and comorbid diseases possess higher severity and mortality in COVID-19 and SARS patients (41). Studies also found that patients with diabetes, heart disease, and other comorbidities had an increased risk of severity (42, 43). Similar to other studies, we also found comorbidity is a prime risk factor for COVID-19 patients in Bangladesh.

The most prevalent symptom in the COVID-19 patient was fever followed by exhaustion, cough, loss of taste, sore throat, and body ache. In general, all the symptoms showed up within 10 days of infection. However, 20% of patients who developed blistering demonstrated onset after 14th days of disease. While the incidence rate is low and the actual mechanism is still unknown, it is possible that angiotensin-converting enzyme-2 (ACE-2) available under the skin may play a role in the dermatological manifestation of SARS-CoV-2 virus can enter through ACE-2 receptors (44). Furthermore, some other neurological symptoms such as Confusion and loss of speech were also developed after the 14th day of infection. Neurological symptoms were found in 36.4% of patients in a previous study conducted in 214 patients (45). Another study conducted in France found that more than two-thirds of the cases demonstrated altered consciousness, including agitation and Confusion (46). Although SARS-CoV-2 can induce neurological damage, the exact mechanism is yet to be discovered. It is postulated that the virus can disseminate through the cribriform plate and olfactory bulb; and damage the capillary endothelium to induce brain injury (45). A preclinical study showed that the SARS virus could cause neuronal loss through transneuronal spreading from the olfactory bulb in K18-hACE2 mice (47). Hence, several reasons such as viral encephalitis, metabolic perturbation, infectious, toxic encephalopathy, seizures with post-ictal Confusion, and stroke could be the underlying reason behind neurological symptoms (46).

While most symptoms persisted for 1 to 10 days, some symptoms such as extreme exhaustion (33%), loss of smell (11%), loss of speech, blistering (20%), and confusion (27%) persisted for more than 14 days. While it is not clear why the onset and duration of symptoms vary from patient to patient, studies suggest that low interferon response in some patients could be the possible underlying mechanism of severity (48, 49). Nevertheless, the onset and duration data also match the study conducted by Lauer et al., where they analyzed 181 cases from outside Hubei province, China, and found the onset of symptoms for SARS-CoV-2 is ~5 days, and 101 out of every 10,000 cases developed symptoms after 14 days of monitoring or quarantine (50).

## Conclusion

This study demonstrates the prevalent symptoms in COVID-19 patients affected from March 2020 to September 2020 in Bangladesh. To date, there are limited data available for the prevalence of symptoms in COVID-19 patients in Bangladesh. This study found that older (>40 years old) and comorbid patients required more hospitalization due to the COVID-19 associated complications, where comorbid patients were more susceptible to have shortness of breath, vomiting, loss of speech, and confusion. In regards of gender difference, the prevalence of sore throat, loss of smell, and vomiting were significantly higher in the male patient. Age and comorbidity also played a crucial role in the duration required to recover from all the COVID-19 associated symptoms. Since this study is highly relevant to the clinical outcomes of COVID-19, the data on different predictors such as age, gender, and comorbidity observed can provide valuable insight into the management of COVID-19 symptoms and can also lead to the understanding of disease progression.

## Data Availability Statement

The raw data supporting the conclusions of this article will be made available by the authors, without undue reservation.

## Ethics Statement

The studies involving human participants were reviewed and approved by Jagannath University Research Cell. The patients/participants provided their written informed consent to participate in this study.

## Author Contributions

MA, MH, and TA conceptualized the study, analyzed the data, developed the methods and materials, and wrote the manuscript. MA and TA conducted the study. MH and TA edited the manuscript. All authors contributed to the article and approved the submitted version.

## Conflict of Interest

The authors declare that the research was conducted in the absence of any commercial or financial relationships that could be construed as a potential conflict of interest.

## Publisher's Note

All claims expressed in this article are solely those of the authors and do not necessarily represent those of their affiliated organizations, or those of the publisher, the editors and the reviewers. Any product that may be evaluated in this article, or claim that may be made by its manufacturer, is not guaranteed or endorsed by the publisher.
