# Supplementary figures and images for: Prevalence of Covid-19 Associated Symptoms, Their Onset and Duration, and Variations Among Different Groups of Patients in Bangladesh
Source: Front Public Health. 2021 Sep 29;9:738352. doi: 10.3389/fpubh.2021.738352 (PMC8511678; doi:10.3389/fpubh.2021.738352)

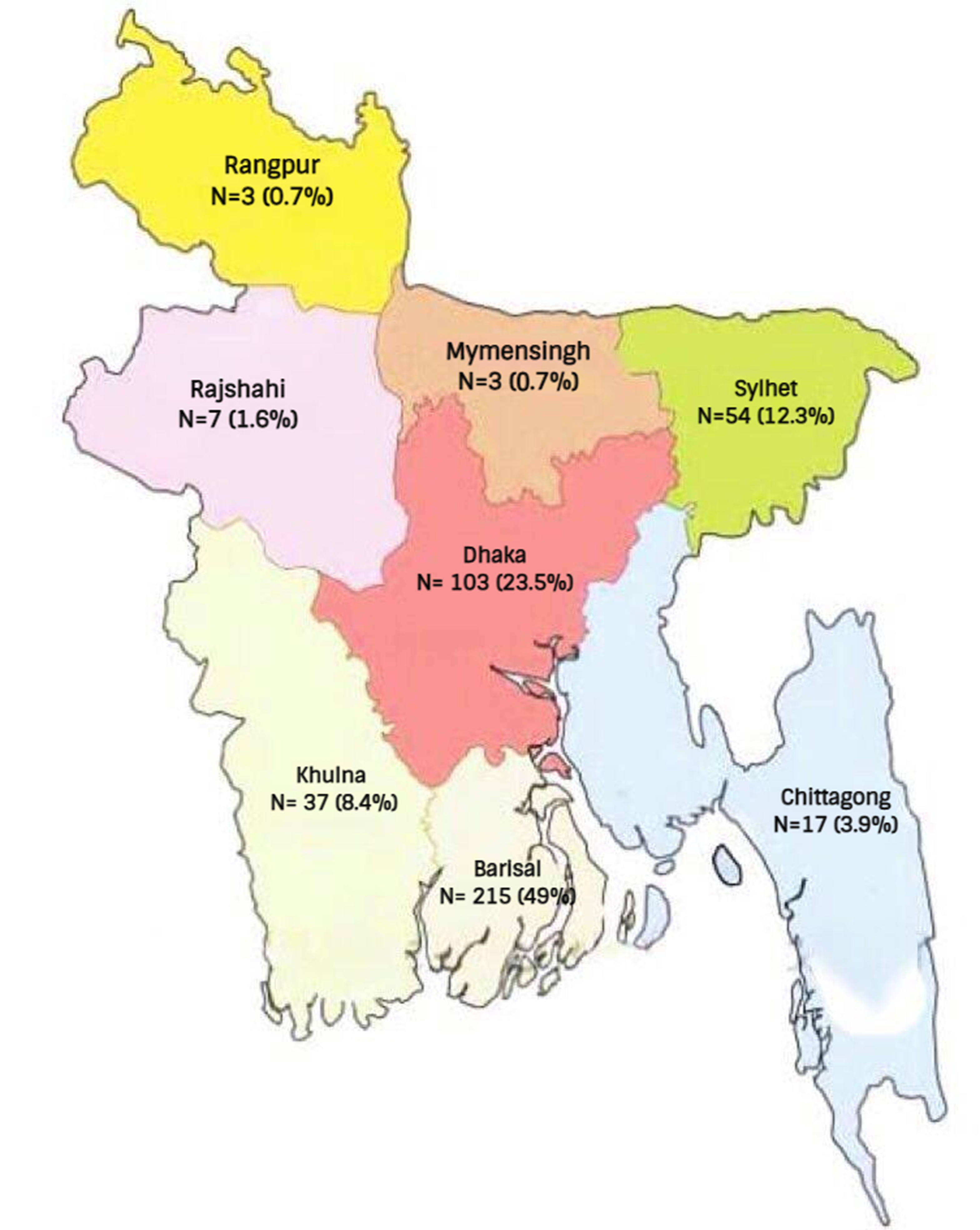

Supplement: Supplementary file 1 [file Image_1.jpg]
